# Editorial: Adolescent brain and alcohol

**DOI:** 10.3389/fphar.2022.1063446

**Published:** 2022-10-28

**Authors:** Subhash C. Pandey, Fulton T. Crews, Susan F. Tapert

**Affiliations:** ^1^ Center for Alcohol Research in Epigenetics, Department of Psychiatry, University of Illinois at Chicago and Jesse Brown VA Medical Center, Chicago, IL, United States; ^2^ Bowles Center for Alcohol Studies School of Medicine, University of North Carolina at Chapel Hill, Chapel Hill, NC, United States; ^3^ Department of Psychiatry, University of California San Diego, La Jolla, CA, United States

**Keywords:** adolescent brain, binge drinking, alcohol use disorder, animal model, brain connectivity

**FIGURE 1:**
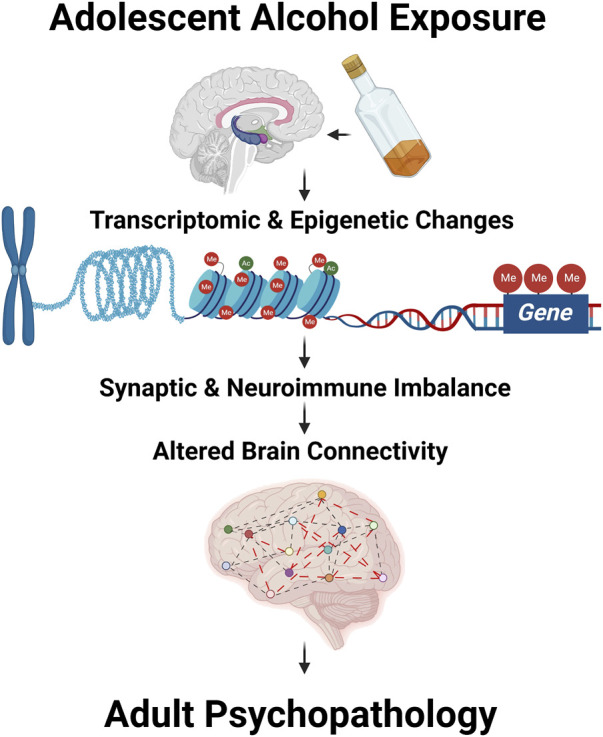
Adolescent alcohol exposure modulates transcriptomic and epigenomic changes leading to altered synaptic and neuroimmune imbalance and abnormal brain structural changes including brain connectivity. These changes together may drive the adult psychopathology after adolescent alcohol exposure.

Binge drinking is prevalent during adolescence and has been shown to increase the susceptibility to adult psychiatric and substance use disorders (Crews et al., 2019; Tapert and Eberson-Shumate, 2022). Adolescence is a critical period for brain maturation, during which several molecular and cellular changes including epigenetic changes take place, leading to synaptic remodeling and pruning (Kyzar et al., 2016; Crews et al., 2019; Tapert and Eberson-Shumate, 2022). Several clinical and preclinical studies (Kyzar et al., 2016; Crews et al., 2019; Tapert and Eberson-Shumate, 2022) in the field clearly suggest that adolescent drinking has long-lasting influences on molecular functioning, brain connectivity, and associated behavioral vulnerability in adulthood (Figure 1). The manuscript and review articles collected in this Research Topic of “*Adolescent Brain and Alcohol*” have summarized recent developments in the field that further advance our understanding of the neurobiology of adult psychopathology after adolescent alcohol exposure.

This Research Topic contains two clinical studies. The first, presented by May et al., provided several interesting findings. First, 24% of 9–10-year-olds in the community sample reported having had at least one sip of alcohol, and had done so 4 times on average, with some as many as 260 times, and had their first sip typically at age 7 (SD = 1.91). Most (76%) alcohol sipping was outside of religious ceremony contexts. Second, support vector machine learning determined that neural activity in brain reward and cognitive control areas (nucleus accumbens and inferior frontal gyrus) was no better than chance at identifying which youth reported alcohol sipping at Time one but was reasonably accurate (76%) when combined with baseline sipping behavior to predict drinking status at Time 2, even after controlling for demographic factors. These studies suggest that improving our understanding of the neural and behavioral factors that convey propensity for future substance use is crucial for identifying at-risk youth and targets for prevention. The second clinical study, presented by Lorkiewicz et al., found that 16% in a community cohort of adolescents and young adults had at least one alcohol-related blackout, and most who did had more than one, with durations exceeding 1 h. Latent growth curve modeling found that having had one or more alcohol-related blackouts was linked to attenuated performance over time on a memory for faces task, which was worse yet when alcohol intake was more frequent, above and beyond effects of age, sex, race, socioeconomic status, and assessment site, and in a model that accounted for repeated testing. Results suggest that alcohol-related blackouts appear to predict lasting changes in visual learning and memory, particularly of a facial nature, suggesting that memory for faces is sensitive to heavy alcohol use and that the late-adolescent/emerging adult brain is vulnerable to alcohol-related blackouts.

This Research Topic also contains five manuscripts that discuss the lasting impact of adolescent intermittent ethanol (AIE) on adult brains by using an underage drinking model in rodents. The review article by Macht et al. covers fetal and adolescent brain development and the impact of alcohol, particularly increases in proinflammatory signaling that modulate cholinergic neurons and hippocampal neurogenesis contributing to lasting pathology in adulthood. Ethanol and stress exposure studies find neuroimmune signaling causes persistent changes that can be reversed. The study described by Gomez et al. use fcMRI to show that AIE disrupts adult synchrony between brain networks related to deficits in behavioral flexibility, extending studies indicating that AIE causes long-lasting changes in adult executive prefrontal cortical functions. Interestingly, studies find fcMRI reductions within a subnetwork of affected brain regions statistically mediated errors committed during reversal learning. These results provide a novel link between persistent reductions in fcMRI brain functional connectivity and adult deficits in behavioral flexibility resulting from AIE. Interestingly, these learning deficits are extended in the studies by Barnett et al. that find AIE accelerates age-related onset of Alzheimer’s disease pathology in a mouse model due to proinflammatory gene induction that can be prevented by blocking microglial activation with minocycline.

Towner et al., investigated the effects of AIE on neuronal activation and social preference using cFos-LacZ transgenic rats and found that AIE produces sex-specific social impairments that are potentially driven by differential neuronal activation states in regions important for social behavior, including the medial prefrontal and orbitofrontal cortices, nucleus accumbens, lateral septum, and central amygdala. Another study published by Chandler et al. investigated the effects of AIE on conditioned fear learning and memory in adulthood in rats. They demonstrated sex-specific changes in conditioned fear behaviors that are reversible by pharmacological interventions (mGlu5 positive allosteric modulator CDPPB) targeting mGlu5 receptor activation. In one manuscript included in this Research Topic, studies conducted by Barney et al. focus on the effects of acute ethanol exposure on the expression of growth factors and microRNA expression profiling in dorsal hippocampus of adolescent and adult rats*.* They concluded that acute ethanol rapidly induced neuroimmune gene expression that was associated with changes in growth factors, in addition to decreasing the expression of several miRNA species in the hippocampus.

Together, these studies emphasized that adolescent alcohol exposure has several negative consequences ranging from molecular to neuronal to circuitry and later, behavioral responses in adulthood. These studies summarize emerging developments in the field’s effort to pinpoint alcohol’s role in adolescent brain producing rapid and long-lasting impacts on neurobiology in adulthood and increasing vulnerability to adult psychopathology including development of alcohol use disorder.
